# Refractory Central Neurogenic Hyperventilation: A Novel Approach Utilizing Mechanical Dead Space

**DOI:** 10.3389/fneur.2019.00937

**Published:** 2019-09-04

**Authors:** Alexander J. Sweidan, Matthew M. Bower, Jeffrey Paullus, Michelle Sterpi, Sara Stern-Nezer, Cyrus Dastur, Wengui Yu, Leonid I. Groysman

**Affiliations:** ^1^Libera Università Campus Bio-Medico di Roma, Rome, Italy; ^2^Department of Neurology, University of California, Irvine, Irvine, CA, United States; ^3^Mount Sinai West, New York City, NY, United States

**Keywords:** mechanical ventilation, lymphoma, central neurogenic hyperventilation, neuro-oncology, neurocritical care, critical care

## Abstract

This report describes the successful management of a case of central neurogenic hyperventilation (CNH) refractory to high dose sedation by increasing the mechanical dead space. A 46-year-old male presented with a history of multiple neurological symptoms. Following an extensive evaluation, he was diagnosed with primary diffuse CNS lymphoma and started on high dose steroids. After initial symptomatic improvement, the patient developed increasing respiratory distress and tachypnea. He was intubated and transferred to the neurointensive care unit (neuro ICU). While in the ICU the patient remained ventilator dependent with significant tachypnea and respiratory alkalosis resistant to fentanyl and propofol. This prompted an attempt to normalize the PaCO_2_ via an increase of the mechanical dead space. This approach successfully increased PaCO_2_ and bridged the patient until ongoing therapy for the underlying disease resolved the pervasive breathing pattern typical of CNH. Further investigation is warranted to evaluate this strategy, which upon review of the literature appears underused.

## Introduction

Central neurogenic hyperventilation (CNH) is a rare condition characterized by hyperventilation that persists even during states of sleep, low PaCO2, and high arterial pH in the absence of toxic or metabolic etiologies. It was first described in patients exposed to acute anoxia by Plum and Swanson (1). It has subsequently been described in several other chronic, diffusely infiltrating processes such as diffusely infiltrating lymphomas, pontine glioma, medulloblastoma, anti-NMDAR encephalitis, multiple sclerosis, histiocytosis, and even a case of laryngeal carcinoma (2, 3). Unfortunately, the pathophysiological mechanisms remain poorly understood and the treatment options remain limited. Here we describe a patient with CNH that was refractory to conventional analgesics and sedatives but responded well to increasing the mechanical dead space.

## Case

**Figure 1:**
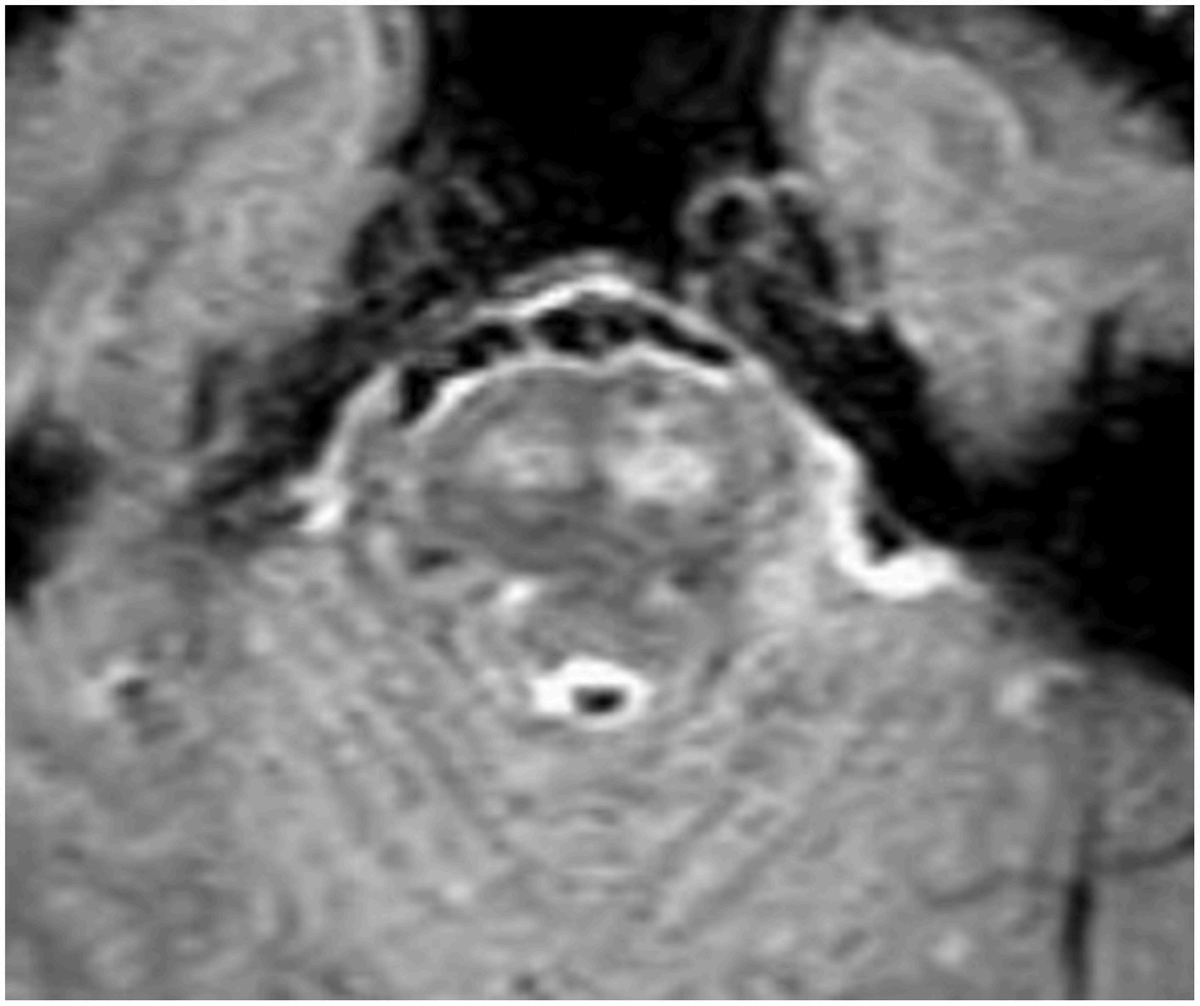
Fluid attenuated inversion recovery (FLAIR) demonstrating pontine hyperintensity from lymphoma involvement.

A 46-year-old male with no prior medical history presented to UC Irvine with a several month history of episodes of right and left hemiparesis, progressive bulbar weakness, paresthesia, dysarthria, and headache. He also reported a 30-pound unintentional weight loss. He was admitted to the neurology service and underwent an extensive evaluation for autoimmune, demyelinating, vascular, and neoplastic processes. MRI brain demonstrated several small discrete foci of restricted diffusion in the white matter, enhancement in large portions of the bilateral corticospinal tracts, and significant pontine involvement (Figure 1). Lumbar puncture demonstrated CSF pleocytosis and elevated protein. Histology of the right frontal brain lesion was consistent with diffuse large B-cell lymphoma (DLBCL), and a bone marrow biopsy demonstrated normocellular marrow. The patient was diagnosed with primary diffuse CNS lymphoma (PCNSL) and started on high dose steroid therapy.

Early in the hospital course the patient was found to be in acute respiratory distress presenting with tachycardia, tachypnea, and increased work of breathing. He was emergently intubated and transferred to the neuro ICU. CT angiogram of the chest was negative for pulmonary embolism but demonstrated interval left lower lobe consolidation. The patient was placed on multiple conventional ventilator modes with a set tidal volume of 400 ml and pressure support of 5 cm H_2_O. Despite these standard settings, large tidal volumes were observed ranging from 700 to 1,400 ml. Initial arterial blood gas (ABG) was significant for a pH of 7.61 and PaCO_2_ of 13.1 mmHg. Due to the significant hypocarbia, a number of ventilator tubing segments were added to increase the mechanical dead space and increase the rebreathing of exhaled CO_2_. In addition, whole brain radiation and treatment with rituximab were initiated. Before these interventions, the patient's neurological exam was poor. He did not follow commands or open eyes to noxious stimuli. However, the patient had intact brainstem reflexes and trace extremity movement to noxious stimuli.

CO_2_ was monitored with ABG and immediate response to the increase in dead space was measured with VBG. Before intervention, VBG showed pH 7.57 and PvCO2 18.4 mmHg which immediately showed signs of improvement after 1 day. Two days after the increase in dead space a follow-up ABG showed pH 7.45 and PaCO_2_ 30.5 mmHg. Over the coming days the patient required ventilator and dead space adjustments as he was weaned off of fentanyl and midazolam to dexmedetomidine and oxycodone.

The patient required a tracheostomy due to prolonged ventilator time, but 2 weeks after the initiation of dead space therapy the patient was weaned from the ventilator with T-piece uncapped. He was off sedation and had been weaned off of opioids. The patient was transferred to the step-down unit with ABG showing pH 7.51 and PaCO_2_ 28.5 mmHg without tachypnea. In those 2 weeks the patient's neurological exam had improved significantly, and he was awake, alert, and attending to the examiner. He followed motor commands in all extremities (distal strength better than proximal strength) and also followed commands to close eyes and stick out tongue. On discharge 1 week later (3 weeks after the increase in dead space) he was smiling and interactive with family.

## Literature Review and Proposed Refractory Management

CNH is a rare neurologic complication first described by Plum and Swanson (1). Diagnostic criteria for CNH include hyperventilation that even during sleep, low PaCO_2_, high PaO_2_, and high arterial pH in the absence of drugs or metabolic causes (3). It is a diagnosis of exclusion, and its true incidence may be underestimated as its presenting signs are often attributed to other conditions which often delays diagnosis.

Several pathophysiological mechanisms have been proposed to explain CNH. In Plum and Swanson's initial report of nine comatose patients, the lesions common to the autopsied cases involved the medial pons, sparing the lateral pontine region, and the medulla oblongata. These findings led the authors to postulate that CNH is the result of an unrestrained stimulation of both the inspiratory and expiratory centers in the medulla via the lateral reticular formation in the pons and the laterally located descending neural pathways (1). However animal models in which the pontine respiratory group was disconnected from the medulla did not produce CNH (4). Bateman et al. on the basis of post-mortem neuropathological studies demonstrating brainstem sparing, further argued against the necessity of a direct destruction of the brainstem respiratory centers for the development of CNH (5).

Another hypothesis posits that the tumor may reduce local pH in the brainstem without altering overall CSF pH, resulting in hydrogen ions stimulating chemosensitive areas located in the ventral brainstem (2, 6). However, this explanation has been challenged as *in vivo* measurements of the pH in brain neoplasms using positron emission tomography (PET) have demonstrated that tumor pH was higher than in the rest of the brain (7). Finally, it has been proposed that the tumor microenvironment cytokines and other neuroactive chemicals may stimulate respiratory centers (2). Animal models have shown that microinjections of glutamate in the brainstem respiratory centers can induce respiration (8). Nevertheless, an unequivocal explanation has not yet been identified and there is still no clear understanding of the pathophysiologic sequence underlying CNH.

CNH is most common in comatose patients and very rare in an awake patients with only about 35 reports in the literature (3). Conversely, the patients who are alert at the onset of CNH are usually so despite very low PaCO_2_ concentration, suggesting that level of consciousness is more dependent on the underlying cause of the CNH rather than the degree of hypocarbia. Cancer-related CNH presents more commonly in a conscious patient. PCNSL accounts for half of such cases according to a recent review (2, 3). About 90% of PCNSLs are DLBCL and 10% are various other lymphomas such as Burkitt's lymphomas or T-cell lymphomas (9). Tumor-induced CNH usually constitutes a presenting symptom as well as an indication of the severity of the underlying disease, as it often precedes the cardiorespiratory deterioration. Prognosis in CNH and especially in CNH secondary to cancer, is usually very poor, with an overall survival after diagnosis of only a few months (3). Hypocapnia alone is unlikely responsible for the increase in mortality or morbidity, but studies of medical and surgical ICU patients with severe alkalemia has shown increased mortality with a rate of up to 80% above pH 7.65 (10, 11). Hypocapnia also leads to undesirable side effects such as impairment of tissue oxygenation (due to left shift of the oxygen dissociation), a decreased cerebral circulation, a resetting of the chemoreceptors and a compensatory distal renal acidosis (12–14).

A unique feature of DLBCL compared with other brain tumors is its unique sensitivity to steroids, although a multi-modality therapy is usually preferred due to the high probability of recurrence in patients treated solely with steroids. Hence the standard of treatment is a combination of corticosteroids, radiation therapy, and chemotherapy in order to increase overall survival and progression free survival (9). As reported above, the patient in our case showed a significant response to IV solumedrol administered shortly after diagnosis and then benefitted from 15 treatments of whole brain radiation and eight doses of weekly rituximab after the onset of CNH. As previously mentioned, treatment directed toward DLBCL ultimately resolved the patient's perverse breathing pattern; however, the patient required bridging therapy.

Management of CNH has been well-described in the literature. In brief, the cornerstone of treatment involves elucidating the underlying cause, treating it, and acutely bridging treatment with high dose sedation, whether it be benzodiazepines, propofol, or analgesia. However, herein we have identified a refractory entity; a patient presenting with an ABG showing pH of 7.61 and PaCO_2_ of 13.1 mmHg which did not respond to fentanyl or propofol. Very few cases have been reported with such refractoriness. One report of a patient with CNH secondary to lymphomatoid granulomatosis whose severe alkalosis was unresponsive to morphine was managed with intubation, mechanical ventilation and paralysis with pancuronium bromide (15). More recently, Jones et al. illustrated that the off-label administration of a single 2 mg/kg dose of ketamine in a patient with a sedation resistant CNH reduced hyperventilation and allowed successful extubation within 24 h (16).

Upon review, however, an increase in mechanical dead space has been scarcely acknowledged as a tool for managing hypocarbia and to our knowledge there have been no reports so far of its successful employment in the setting of CNH. The ventilator circuit is a mechanical dead space which is a frequently forgotten. Increased mechanical dead space facilitates greater rebreathing of exhaled PaCO_2_. Suwa et al. investigated the feasibility of restoring a low PaCO_2_ to normal by the addition of a mechanical dead space without changing the pattern of ventilation. Their population consisted of long-term mechanically ventilated patients, and they were able to formulate an equation to predict the increase in PaCO_2_ (12). Pauzner et al. mentioned in their case report a 2-h trial of breathing through increased dead space as one of the first attempts to control a resistant episode of tumor-induced CNH. Ultimately the patient responded to multiple doses of morphine that were given only after the failure of the aforementioned increase in dead space (17).

In our patient, a number of ventilator tubing segments were added to increase the mechanical dead space and increase the rebreathing of the exhaled CO_2_. The mechanisms involved in this are beyond the scope of this review; a simple heuristic, for every 100 mL (1 ft or 30.5 cm) of circuitry added the PaCO_2_ increases ~5 mmHg. We added ~6 ft of mechanical dead space to our patient's circuitry. The patient did not have an underlying lung disease. Therefore, this temporizing bridging modality was effective enough to wean the patient to 6 ft of mechanical T-piece dead space. This technique maintained a healthy pH, PaCO_2_, and PaO_2_ for ~2 weeks. As the patient's DLBCL became increasingly responsive to radiation and high dose steroids, the mechanical dead space was gradually weaned with stable ABG parameters.

The advantages of applying such a method derive from its roughly predictable results and minimal collateral effects, as well as the opportunity it may offer to administer smaller dosages of sedating pharmaceuticals in already compromised patients. These benefits and the relative low cost of this technique should warrant further investigations into the timing, sequencing, and optimal integration with the other measures employed in the management of CNH.

## Conclusion

We report a patient with a central neurogenic hyperventilation secondary to a diffuse large B cell lymphoma resistant to high sedation. The refractory alkalemia was managed by treating the underlying lymphoma and bridging therapy with an increase of the mechanical dead space through the addition of ventilator tubing segments. The combination of these measures normalized the PaCO_2_ and pH, allowing the patient to be weaned off the ventilator. To our knowledge there have been no other successful employments of this technique in stabilizing patients during episodes of this abnormal breathing pattern.

## Ethics Statement

Written informed consent was obtained from the participant/patient for both participation and publication of the case report.

## Author Contributions

AS and MB: drafting, revising, and finalizing the manuscript. All others: reviewed and revised critically for important intellectual content.

### Conflict of Interest Statement

The authors declare that the research was conducted in the absence of any commercial or financial relationships that could be construed as a potential conflict of interest.

## Abbreviations

CNH: central neurogenic hyperventilation
DLBCL: diffuse large B-cell lymphoma
PCNSL: Primary CNS lymphoma
ABG: arterial blood gas
Neuro ICU: neurointensive care unit.
